# Multifunctional Pd-Based Nanocomposites with Designed Structure from In Situ Growth of Pd Nanoparticles and Polyether Block Amide Copolymer

**DOI:** 10.3390/polym13091477

**Published:** 2021-05-03

**Authors:** Kevin Dal Pont, Anatoli Serghei, Eliane Espuche

**Affiliations:** Univ Lyon: UCBL, CNRS, IMP UMR 5223, Ingénierie des Matériaux Polymères, F-69622 Villeurbanne, France; kevin.dalpont@gmail.com (K.D.P.); anatoli.serghei@univ-lyon1.fr (A.S.)

**Keywords:** nanocomposites, copolymer, palladium nanoparticles, conductive material, hydrogen solubility

## Abstract

Nanocomposites containing palladium nanoparticles were synthesized by in situ generation route from palladium acetate and a polyether block amide matrix with the aim to obtain materials with specific nanoparticle location and function properties. The chosen Pebax matrix was composed of a continuous soft phase containing dispersed semi-crystalline rigid domains. Nanocomposite films with Pd amount up to 30 wt% (corresponding to 3.5 vol%) were directly prepared from the palladium precursor and the copolymer matrix through a solvent cast process. The microstructure of the films was investigated by microcalorimetry, X-ray diffraction analyses and transmission electron microscopy. The nanocomposites’ function properties in terms of electrical conductivity and interaction towards hydrogen were studied as a function of the palladium content. It was shown that the spherical crystalline Pd nanoparticles that were in situ formed were located in the continuous soft phase of the copolymer matrix. They did not induce modification of Pebax microstructure and chain mobility. The specific location of the metal nanoparticles within the copolymer matrix associated with their low size allowed obtaining conductive materials for Pd amount equal to 3.5 vol%. Moreover, the affinity towards hydrogen evidenced from hydrogen permeation experiments made this nanocomposite series promising for further development in sensing applications.

## 1. Introduction

Organic–inorganic composites have attracted great attention because of their potential to combine the features of organic materials with those of inorganic materials. Particularly, polymer–metal nanocomposites are promising functional materials in domains such as optical, electrical, magnetic, catalytic and gas transport applications [1,2,3,4,5,6]. Such nanocomposites can be prepared either by the ex situ method or by the in situ generation route. 

The ex situ method consists of dispersing preformed metal nanoparticles within the polymer matrix [7,8,9,10,11,12]. The metal fillers usually consist of elementary nanoparticles forming agglomerates. To reach dispersion at the nanometre scale, particle–particle interactions must be broken by thermomechanical strain. Moreover, modification of the filler and/or the polymer matrix must often be carried out to promote filler/matrix interactions [13,14]. Despite these approaches, the dispersion of individual nanofillers is often difficult to achieve [14,15]. In addition, another obstacle to the development of such nanocomposites preparation route is the increasing concerns about nanoparticle manipulation [16]. 

The in situ method is an alternative way to prepare nanocomposites [2,3,4,6,17,18,19,20,21,22]. In that case, a metal precursor is usually dissolved in a polymer solution. To grow the noble metal nanoparticles, the reduction of the metal precursor has to take place either in the polymer solution or in the polymer film obtained after smooth evaporation of the solvent. Depending on the polymer/metal precursor system, different ways can be used for the metal ion reduction step. The most common way consists of using an external reducing agent [4,6,20,23], such as sodium borohydrate as an example. Thermal annealing or UV-irradiation have also been reported as efficient ways to obtain polymer-embedded metal nanoparticles for some polymer/metal complex systems [3,18,19,21,22,24,25]. The in situ generation route generally allows avoiding nanofiller aggregation. However, one drawback of this route is that the amount of generated metal is generally low (often less than 15 wt% corresponding to low volume % of metal nanoparticles) due to embrittlement at higher filler loading. This low filler amount cannot allow one to reach the percolation threshold that is necessary to obtain conductive nanocomposites. Indeed, the percolation threshold is obtained around 30 vol% for spherical conductive fillers dispersed in a homogeneous polymer matrix.

Several studies performed on nanocomposites obtained by dispersion of preformed nanofillers have shown that it was possible to decrease the filler amount needed to reach the percolation threshold by using immiscible polymer blends instead of homogeneous polymer matrices [26]. To be efficient, the polymer blend morphology has to be co-continuous, meaning that the blend components and composition must be carefully controlled. Moreover, the fillers have to be exclusively located in one phase of the blend. 

The aim of the present work was to reach such a morphology by combining in situ grown nanoparticles and a polyether block amide copolymer matrix. This copolymer family which is industrially available under the trade name Pebax^®^ belongs to the thermoplastic elastomers (TPEs) family [27,28,29]. Due to the association of one block which acts as the hard phase with one block which acts as the soft phase, TPEs offer the advantage of exhibiting high extensibility and elasticity at their service temperatures. They can be processed as thermoplastics, and they exhibit a microphase separation morphology in the solid state because of the high polarity difference between their hard and soft segments. This phase separation should then be advantageously exploited to obtain a preferential location of the metal nanoparticles in the continuous soft phase, allowing reaching the percolation threshold at a lower filler amount and obtaining conductive nanocomposites while maintaining flexibility. 

Among the wide range of metal precursors that could be used, we chose to work with a palladium precursor. Indeed, palladium is of great interest due to its catalytic properties and its specific affinity towards hydrogen that could be applied for hydrogen separation, fuel cell membranes or hydrogen sensing [4,5,6,17,18,19]. Synthesising nanocomposites able to combine electrical conductivity and specific interactions towards hydrogen through the original synthesis route and controlled architecture could then be of great interest for developing a new range of function materials. 

The aim of this work was to develop multifunctional Pd-based nanocomposites by exploiting the in situ generation route of nanoparticles and the specific morphology of polyether block amide copolymer. According to our knowledge, no work based on such an approach could be found in the literature. 

## 2. Materials and Methods

### 2.1. Materials and Nanocomposite Preparation 

#### 2.1.1. Materials Used

The polyether block amide, Pebax^®^ MV3000, was kindly supplied by Arkema Inc. (Serquigny, France) in the form of pellets. It is a thermoplastic elastomer made of a flexible polyether and rigid polyamide whose density is 1.02 g·cm^−3^. Palladium acetate (PdAc_2_, purity higher than 99%) was purchased from Fluka (Saint Quentin Fallavier, France) and 1-butanol (MC, grade for analysis) from Acros Organics (Belgium). 

#### 2.1.2. Film Preparation

Reference copolymer films were prepared by dissolving the Pebax^®^ MV3000 pellets in 1-butanol at 90 °C and stirring overnight in order to obtain a homogeneous solution with a 2 wt% polymer concentration. The solution was cast in Teflon-coated Petri dishes. After smooth evaporation of the solvent at room temperature, a final thermal treatment at 70 °C for 2 h was applied in order to obtain films free of residual solvent.

To prepare the nanocomposite films, different amounts of palladium acetate were first dissolved in butanol at room temperature. The PdAC_2_ solution was progressively poured into the polymer solution, and further stirring was performed for 1 h to ensure complete mixing. A colour change from transparent to dark colour was observed showing that the reduction of the Pd precursor occurred during this step. The solutions were then cast in Teflon-coated Petri dishes. The nanocomposite films were obtained after smooth evaporation of the solvent at room temperature followed by the final thermal treatment of 2 h at 70 °C. 

The reference copolymer films were denoted P0, and the nanocomposite films were named P-x Pd, where x corresponds to the weight % of Pd contained in the film. x was increased up to 30 wt%, corresponding to a volume fraction of 3.5 vol%. The Pd volume fraction was calculated by taking a density value equal to 12.02 g·cm^−3^ for Pd. The prepared films were 70 μm thick.

### 2.2. Film Characterization 

#### 2.2.1. Differential Scanning Calorimetry (DSC) 

Differential scanning calorimetry (DSC) analysis was performed with a TA Instruments Q200 (Waters Corporation, Milford, MA, USA) apparatus under helium atmosphere at a scanning rate of 10 °C/min from −80 °C to 180 °C. Two scans were successively recorded with intermediate cooling at 10 °C/min. The uncertainty on the characteristic transition temperatures was ±1 °C, and the uncertainty on the enthalpy values was 5%.

#### 2.2.2. Thermal Gravimetry Analysis (TGA) 

Thermal gravimetry analysis (TGA) was performed with a TGA apparatus (TGA 2950-TA Instruments, Waters Corporation, Milford, MA, USA) under helium atmosphere. The samples (about 10 mg) were heated from 30 °C to 700 °C at a heating rate of 10 °C/min. The temperature corresponding to 5% weight loss, denoted as T_5%_, was taken as the degradation temperature. The residue at high temperature allowed us to check that the experimental contents of Pd nanoparticles corresponded to the theoretical ones.

#### 2.2.3. Transmission Electron Microscopy (TEM) 

Transmission electron microscopy was performed to investigate first the morphology of the copolymer and then the dispersion state of the metal particles within the copolymer matrix. The films were microtomed at −80 °C with a Leica EMFS instrument equipped with a diamond knife in order to obtain ultrathin sections of about 80 nm thickness. They were then imaged in a Philips CM120 (Philips, Amsterdam, The Netherlands) transmission electron microscope with an accelerating voltage of 80 kV without staining.

#### 2.2.4. Wide Angle X-ray Diffraction (XRD) 

The copolymer microstructure and the metal nanoparticles crystalline structure were investigated at 25 °C by wide-angle X-ray diffraction. The experiments were performed between 5° and 50° by step of 0.02° using a Cu tube (*λ* = 1.5406 Å) and a Bruker D8 advance diffractometer, where the kβ line was removed with a nickel filter. The films were deposited on neutral monocrystalline substrates with a thin transfer adhesive with low scattering response.

#### 2.2.5. Gas Permeability 

Gas permeation experiments were performed at 25 ± 1 °C for hydrogen on samples whose effective area was 3 cm^2^. Preliminary high vacuum desorption was realized during at least 12 h to ensure that the static vacuum pressure changes in the downstream compartment were negligible in comparison with the pressure changes due to the gas diffusion. The gas was introduced in the upstream compartment of the permeation cell under a pressure of 3 bars. The pressure variations in the downstream compartment were recorded as a function of time with a 10 Torr Datametrics pressure sensor. A steady-state line was obtained after a transitory state. The permeability coefficient, *P*, expressed in barrer unit (1 barrer = 10^−10^ cm_STP_^3^ cm cm^−2^ s^−1^ cm_Hg_^−1^ = 3.36 10^−16^ mol m m^−2^ s^−1^ Pa^−1^) was calculated from the slope of the steady-state line. The time lag, *t_L_*, was determined by the intercept of the steady-state line with the time axis. The apparent diffusion coefficient, *D*, was calculated from the following equation:

*D* = *e*^2^/6*t_L_* (1)

where *e* is the sample thickness.

The apparent solubility coefficient, *S*, was deduced from Equation (2) assuming that the transport mechanism obeyed a Fickian law.

*P* = *DS* (2)

The reported results were the average values of measurements performed on three different samples. The deviation on the permeability coefficient and on the time lag values was less than 5%.

#### 2.2.6. Electrical Analysis 

The measurements of the electrical conductivity were carried out between 10^6^ Hz and 1 Hz using a high-resolution dielectric spectrometer (Novocontrol GmbH, Montabaur, Germany). The studied films were gold-coated on their both surfaces using a Sputter Coater (Cressington 108, Cressington Scientific Instruments UK, Watford, UK) in order to eliminate the influence of the contact resistance on the conductivity measurements. The specific measurement area, determined by the geometry of the gold-metallized electrodes, was 0.785 cm^2^, corresponding to a sample diameter of 1 cm. The applied voltage was 0.1 V. 

## 3. Results

The microstructure of reference Pebax films was studied before investigating the palladium/copolymer nanocomposites morphology. The functional properties of the nanocomposites were then discussed with respect to the properties of the reference Pebax samples.

### 3.1. Microstructural Analysis of the Reference and Nanocomposite Films 

#### 3.1.1. Reference Pebax Films

**Figure 1:**
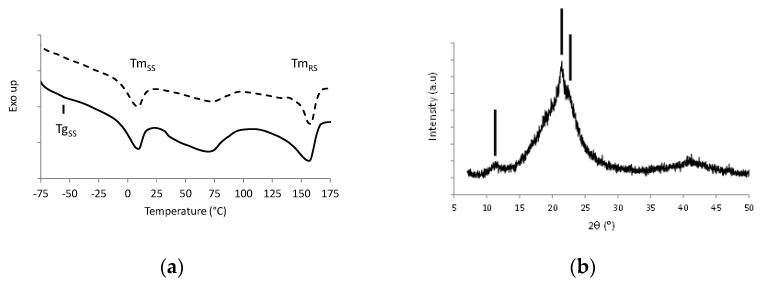
(**a**) DSC first heating scan (full line) and second heating scan (dotted line) of Pebax^®^ MV3000 film; (**b**) XRD pattern of Pebax^®^ MV3000 film (the lines indicate the diffraction peaks at 11.3°, 21.4° and 22.4°, respectively)

Figure 1a shows the DSC thermograms of the reference Pebax film, and Figure 1b represents the XRD diffractogram of the film obtained at room temperature. 

The first DSC heating scan exhibited the specific signatures of the two phases coexisting in Pebax^®^ MV3000, in accordance with its copolymer nature. The glass transition temperature of the polyether soft segments (Tg_SS_) was observed at −59 °C followed by their melting peak at Tm_SS_ around 7 °C. The melting peak of the polyamide rigid segments, Tm_RS_, could be observed at 155 °C. The melting enthalpy values were equal to 16.6 J/g and 28.1 J/g for the soft phase and rigid phase, respectively. The broad endotherm observed in the temperature range between 50 °C and 100 °C could be assigned to the presence of water absorbed within the film due to its hydrophilic nature [30]. The second scan underlined a decrease of this endotherm without any modification of the other signals. 

The XRD diffractogram of the copolymer shown in Figure 1b exhibited an amorphous halo and three reflection peaks at 11.3°, 21.4° and 22.4°. The diffraction peak located around 11° could be assigned to (002) reflection of PA6 γ-phase, and the diffraction peak at angular position 21.4° could be attributed to (020) and (200) reflections of the PA6 γ-form crystals [31]. Two small shoulders associated with (200) and (020) reflections of the crystalline PA6 α-phase were also distinguished at angular position 20.5° and 22.4°, respectively [31]. No diffraction peak related to a PEO-type crystalline microstructure was observed on XRD patterns of the reference Pebax which was in accordance with the preliminary results obtained by DSC and more specifically with the melting of the soft segments at a temperature below 25 °C.

**Figure 2:**
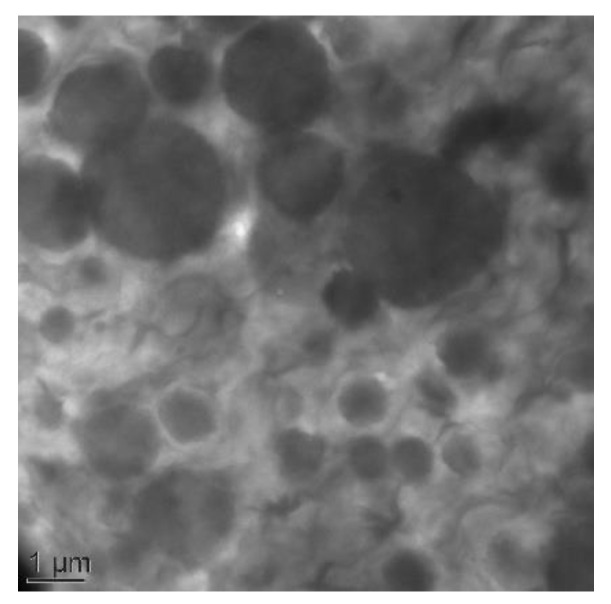
TEM images of Pebax^®^ MV3000 reference films.

The copolymer morphology was observed thanks to TEM analysis. Figure 2 clearly shows the presence of nodular micrometre-sized domains that could be assigned to the semi-crystalline rigid domains dispersed within the continuous amorphous soft phase.

#### 3.1.2. Nanocomposite Films

**Figure 3:**
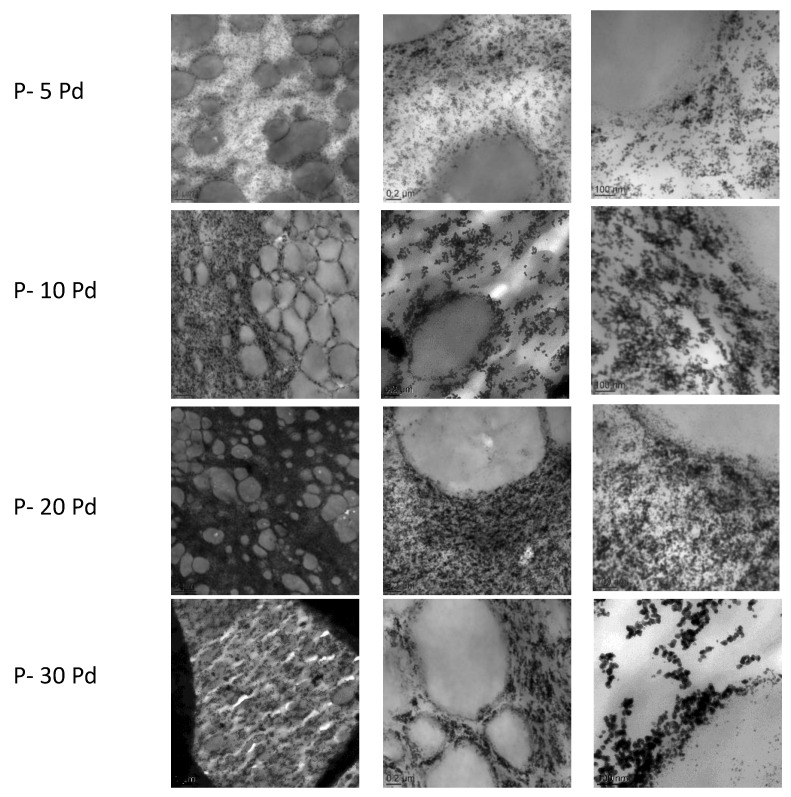
TEM analysis of the nanocomposites at different scales: left, 2μm bar scale; middle, 0.2 μm bar scale; right, 100 nm bar scale.

Figure 3 shows the TEM images representative of the cross-sections of the nanocomposites’ films for increased Pd amount. Pd appeared as dark spherical nanoparticles. Whatever the nanocomposite composition, the nanoparticles were located within the continuous soft phase and totally excluded from the rigid domains of the copolymer. This morphology could probably be explained by the semi-crystalline nature of the copolymer rigid phase. Indeed, a previous analysis performed on PVA showed that metal nanoparticles obtained by in situ growing developed in the PVA amorphous domains [21]. In our case, as the solvent evaporation occurred and Pebax rigid segments organized in crystalline domains, the palladium nanoparticles were discarded from these domains composed of more rigid and densely packed chain segments. They were then preferentially located in the continuous soft phase composed of the amorphous and flexible polyether chains. It could be observed that increasing the Pd amount within the copolymer up to 20 wt% Pd led to an increase in the nanoparticle amount without a major change in the nanoparticle size. For 30 wt% of Pd, the contrast between the metal domains and the polymer matrix seemed to be emphasized and the domains appeared to have slightly higher size while still being in the nanometre scale range.

**Figure 4:**
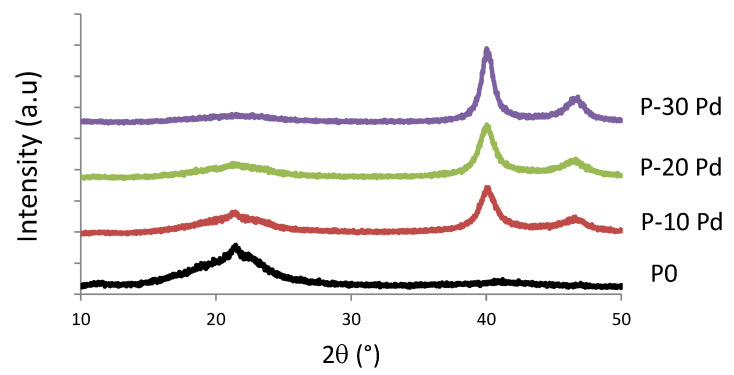
XRD patterns of the nanocomposite films.

The crystalline nature of the in situ grown Pd nanoparticles was checked by XRD analysis. The XRD patterns of the nanocomposites shown in Figure 4 exhibited two well-defined peaks at 40.1° and 46.5 ° in addition to the characteristic response of the copolymer matrix. Each diffraction line could be assigned to a diffraction peak characteristic of fcc metallic Pd (namely (111) diffraction peak at 2θ = 40.1° and (200) reflection at 2θ = 46.5°) [19].

**Figure 5:**
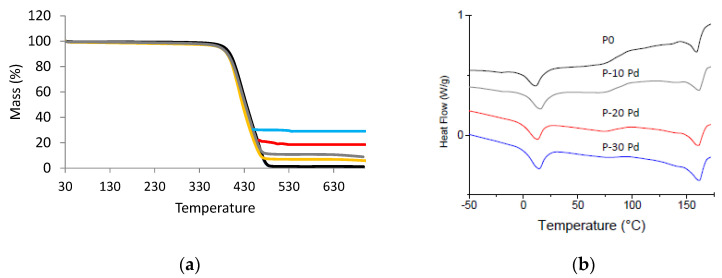
(**a**) TGA curves of the nanocomposites (P0: black; P-5 Pd: yellow; P-10 Pd: grey; P-20 PD: red, P-30 Pd: blue); (**b**) DSC heating scan of the nanocomposites (shifted curves—exo up).

The Pd amount contained in the nanocomposites was experimentally measured thanks to TGA analysis (Figure 5a). The values of the residues at high temperature reported in Table 1 were in good agreement with the theoretical metal contents. Moreover, the degradation temperature of the films, T_5%_, corresponding to a weight loss of 5% was not modified by the presence of Pd nanofillers. It was around 385 ± 2 °C.

The microstructure of the Pebax matrix was investigated for the different nanocomposites with the aim to see if the presence of Pd could modify chain mobility or crystallinity. Figure 5b shows the DSC thermograms obtained for the nanocomposites, and Table 1 summarizes the values of the temperatures of characteristic transitions as well as the values of the melting enthalpy related to the soft and rigid phases, respectively.

The specific signatures of the copolymer soft phase and rigid phase were observed on the DSC thermogram of each nanocomposite. From the values of the characteristic temperatures and melting enthalpy measured for each sample, it could be concluded that the presence of Pd nanoparticles did not significantly modify the copolymer microstructure and chain mobility (Table 1). Indeed, no significant modification of Tg_SS_, Tm_SS_ and Tm_RS_ was observed on the nanocomposites with respect to the neat copolymer. Moreover, the enthalpy values related to the single polymer phase calculated from the enthalpy values reported in Table 1 were in the same range as those measured for the neat Pebax (in the range 26.7–32.5 J·g_Pebax_^−1^ for ΔHm_RS_ and 16.6–19 J·g_Pebax_^−1^ for ΔHm_SS_).

According to the results obtained in this material characterization step, it seemed that the presence of Pd nanoparticles, even at high content, did not modify the microstructure of the Pebax matrix. Thus any evolution of the function properties going from reference Pebax film to nanocomposite films should only be related to the sole effect of the metal nanoparticles.

### 3.2. Function Properties

In this part, we focus on the evolution of the electrical and hydrogen transport properties as a function of the nanocomposites’ metal content.

#### 3.2.1. Electrical Properties

**Figure 6:**
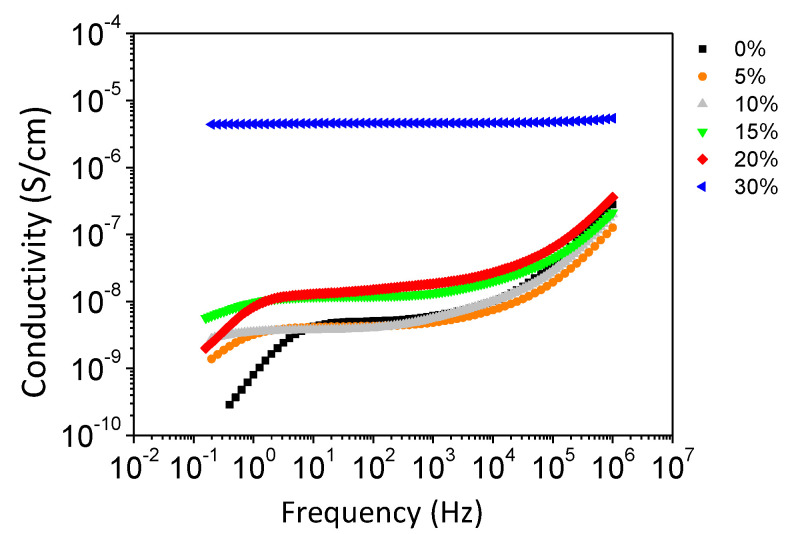
Evolution of the electrical conductivity as a function of the frequency.

The results of the electrical investigations on our nanocomposite materials are presented in Figure 6, showing, at different filler concentrations, conductivity spectra of σ’ (the real part of the complex conductivity function σ* = σ’ + iσ’’).

The nanocomposite materials up to a filler weight concentration of 20% exhibited a typical response corresponding to an ionic conductivity, with a decrease in σ’ detected in the low-frequency spectral region. This decrease was due to electrode polarization effects [32] that originated from a blocking effect of ions at the interface with the measurement electrodes. The ionic conductivity, determined by the plateau value measured in σ’, did not change much with the addition of fillers, indicating the fact that the ionic transport process originated from the pure polymer matrix. Indeed, a value of 5.1 × 10^−9^ S/cm was measured for the pure polymer, in agreement with data reported in the literature [33,34]. A slight increase in the ionic conductivity was observed for a filler concentration of 15 wt% and 20 wt% presumably due to a larger excluded volume related to the presence of fillers. For the nanocomposite material with a filler concentration of 30 wt%, an electronic conductivity of 5.1 × 10^−6^ S/cm was measured in the spectra of σ’. This was due to the percolation of fillers, leading to conductive pathways in the volume of the investigated material. As expected, the percolation of fillers led to a much higher conductivity value (of electronic nature), as compared to the ionic conductivity of the non-percolated composite materials. It was thus inferred that the percolation threshold of the investigated composite materials lay between 20 wt% (corresponding to 1.7 vol%) and 30 wt% (corresponding to 3.5 vol%) of fillers. The Pd volume fraction leading to the percolation threshold was relatively low considering the spherical shape of the Pd nanofillers. It is noteworthy that the copolymer heterogeneous structure associated with the lonely location of the nanoparticles within the copolymer continuous soft phase could explain such a low percolation threshold value. The trends we observed were in good agreement with those obtained by Rybak et al. [26]. These authors worked on conductive composites based on immiscible polymer blends forming co-continuous networks in which preferential location of the fillers took place. The electrical conductivity was controlled by the double percolation conditions, meaning reaching the continuity of the conductive phase within the material and creating the conductive path through the conductive fillers. Reaching these conditions allowed the percolation threshold to decrease by around 8 vol% for HDPE/PBT/Ag composites. It has to be noted that the mean size of the Ag nanoparticles used to prepare HDPE/PBT/Ag composites was 150 nm, thus much higher than our Pd nanoparticles. It seemed then that using smaller-sized conductive particles combined with their specific location in the continuous soft phase of the Pebax copolymer allowed us to reach the percolation threshold at lower filler volume content.

#### 3.2.2. Hydrogen Transport Properties

Hydrogen transport properties were determined at 25 °C for the reference and the nanocomposites films. H_2_ permeability coefficient was equal to 9.6 ± 0.5 barrer for the reference Pebax film, with H_2_ diffusion coefficient being equal to 6.9 ± 0.4 10^−7^ cm^2^·s^−1^ and H_2_ solubility 0.14 ± 0.01 10^−2^ cm^3^·cm^−3^·cm_Hg_^−1^. The rather high level of hydrogen permeability observed on the reference Pebax film with respect to polyamide-type polymers could be assigned to the presence of the continuous rubbery polyether phase which allowed a high hydrogen diffusion rate. The low value of H_2_ solubility well agreed with the low value of the critical temperature of this gas and the absence of specific H_2_/polymer interactions.

The hydrogen transport behaviour of the nanocomposites was investigated with a particular focus on the unsteady-state gas transport properties (especially the time lag value, ***t_L_***) as a means to investigate interactions towards hydrogen. Indeed, Pd is known to be interactive towards hydrogen. The hydrogen transport coefficients are summarized in Table 2.

It has to be noted first that it was possible to successfully perform permeability analyses for Pebax-based nanocomposites containing a Pd amount of up to 30 wt% as these samples retained their flexibility and did not become brittle. Previous work devoted to in situ generation of metal nanoparticles within homopolymers showed that it was difficult to increase the metal nanoparticles’ amount above 15 wt% due to embrittlement [19,20].

The copolymer structure of Pebax with its rubbery continuous phase containing the nanoparticles allowed the nanocomposite films to keep high enough ductile behaviour to mechanically resist a gas pressure of 3 bar.

From the data of Table 2, it could be observed that hydrogen permeability remained rather constant for Pd amount up to 15 wt% and then only slightly decreased. The time lag value increased significantly as the amount of Pd nanoparticles increased within the material. The increase factor was around 40 going from neat Pebax to the nanocomposite film containing 30 wt% of Pd nanoparticles. This led to a significant decrease in the apparent diffusion coefficient calculated according to equation 1. These results confirmed that the Pd nanoparticles contained in the Pebax matrix could entrap hydrogen, leading to a transient state (***t_L_***), during which no hydrogen molecules could diffuse out of the films. This behaviour led to a decrease in the apparent diffusion. Once the entrapment capacity of Pd nanoparticles was exhausted, hydrogen diffused as in a standard nanocomposite film containing non-interactive fillers. Thus the only effect of the nanofillers in the steady state was related to their volume content which remained low (less than 3.5 vol%) and their aspect ratio, both playing a role in tortuosity. As the fillers were spherical, the tortuosity effect was low and the gas permeability only slightly decreased for the higher filler amounts.

The apparent hydrogen solubility calculated from hydrogen permeability and diffusion according to equation 2 increased in a significant way (by a factor around 280 for Pd amount ranging from 0 to 30 wt% Pd (corresponding to 3.5 vol%)), showing the interest of this material series for selective hydrogen entrapment.

**Figure 7:**
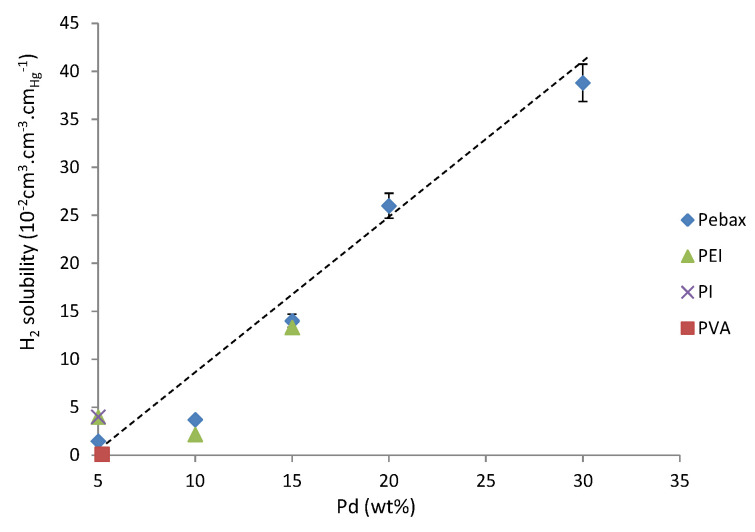
Hydrogen solubility as a function of the Pd nanoparticle content within different polymer matrices [17,19,22]; dotted line is guide for eyes.

The values of hydrogen solubility measured for the current series were compared in Figure 7 with those reported in the literature for nanocomposites prepared from in situ generation routes but from different polymer matrices [17,19,22]. The polymers differed in particular by their hydrogen permeability ranging from P_H_2__ = 0.3 barrer for poly(vinyl alcohol) to 1.2 barrer for polyimide, 5.8 barrer for poly(etherimide) and 9.8 barrer for Pebax.

It could be observed from Figure 7 that the hydrogen solubility increased as a function of the nanoparticle amount. Moreover, it seemed that the polymer matrices and their permeation properties did not play a major role in the range investigated.

## 4. Discussion

The developed nanocomposite series prepared by in situ generation route of Pd nanoparticles allowed us to reach higher amounts of metal nanoparticles than those commonly reported in the literature using the same palladium precursor [19,22]. This could be related to the rubbery behaviour of our copolymer matrix which allowed avoiding material embrittlement at high Pd content. Due to its copolymer nature and specific morphology, our Pebax matrix allowed also obtaining a specific location of the Pd nanoparticles. The nanoparticles were excluded from the semi-crystalline rigid domains, and their location within the continuous copolymer soft phase permitted obtaining nanocomposites with an electrical conductivity value reaching 5.1 × 10^−6^ S/cm for 30 wt% Pd content corresponding to 3.5 vol% nanofillers. According to our knowledge, such a conductive behaviour was never mentioned in the literature concerning Pd-based nanocomposites prepared from such an in situ generation route. The percolation threshold taking place at a very low filler amount (in the range between 1.7 and 3.5 vol% nanofillers) considering spherical nanoparticles could be related to the particles’ low size and specific location.

Another major interest of the developed nanocomposite series was their ability to entrap hydrogen. A linear increase of H_2_ solubility as a function of the Pd amount was obtained. The comparison of the entrapment properties of our nanocomposites series with respect to those reported in the literature [17,19,22] showed that the higher gas permeability of the Pebax matrix with respect to PVA or PEI matrices, due to the rubbery state of the Pebax continuous soft phase, did not impact the H_2_ entrapment ability of the embedded Pd nanoparticles. The use of Pebax matrix allowed us to increase the Pd amount to a higher extent than what was commonly done in the literature while avoiding material embrittlement. As a consequence, it was possible to cover with our nanocomposites series a larger range of hydrogen solubility.

Finally, the series based on Pebax matrix which allowed going from non-conductive to electrically conductive materials while covering a wide range of hydrogen solubility and maintaining material flexibility appeared as particularly interesting for designed functional materials.

## 5. Conclusions

In this work, polymer/metal nanocomposites films were successfully prepared from palladium acetate and Pebax copolymer by using in situ generation route. The in situ grown Pd nanoparticles were crystalline and exhibited a spherical shape. For the whole range of Pd amount investigated (from 0 to 30 wt% corresponding to 0–3.5 vol% Pd), the metal nanoparticles were exclusively located in the continuous soft phase of the copolymer matrix. This specific particle location associated with their nanometre size allowed obtaining electrically conductive materials for a Pd amount as low as 3.5 vol%. Moreover, all materials retained their flexibility. The Pd nanoparticles gave the nanocomposites a specific hydrogen sorption capacity which linearly increased as a function of the Pd amount. The high gas permeability of the copolymer matrix, related to its continuous rubbery polyether soft phase, did not impact the hydrogen entrapment ability of the Pd nanoparticles compared to literature data. The possibility to increase the Pd amount within the copolymer matrix without detrimental impact on the materials’ flexibility permitted, thus, to prepare mechanically stable nanocomposites with enlarged H_2_ sorption ability. Finally, the nanocomposites series based on Pebax matrix and in situ grown Pd nanoparticles which allowed going from non-conductive to electrically conductive materials while covering a wide range of hydrogen solubility appeared as particularly interesting for further studies as sensing materials.

## Figures and Tables

**Table 1:** Residue measured at high temperature from TGA curves and thermal transitions and related enthalpy values measured by DSC; uncertainty on temperature values ±1 °C and on enthalpy values ±5%.

Sample	Residue (%)	Tg_SS_ (°C)	Tm_SS_ (°C)	ΔHm_SS_ (J/g)	Tm_RS_ (°C)	ΔHm_RS_ (J/g)
P0	0	−59	−7	16.6	155	28.1
P-5 Pd	4.8	−60	−9	15.8	153	27.4
P-10 Pd	9.8	−57	−9	15.2	157	25.8
P-20 Pd	19.5	−56	−8	14.1	154	23.6
P-30 Pd	29.3	−55	−7	13.3	156	22.7

**Table 2:** Hydrogen transport coefficients: permeability coefficient, time lag, apparent diffusion coefficient, apparent solubility coefficient measured on reference Pebax films and nanocomposite films (films’ thickness 70 μm).

Sample	*P_H_*_2_ (Barrer)	*t_L_* (s)	*D_H_*_2_ (10^−7 ^cm^2^·s^−1^)	*S_H_*_2_ (10^−2^ cm^3^·cm^−3^·cm_Hg_^−1^)
P0	9.6	11.8	6.9	0.14
P-5 Pd	9.7	12.3	6.6	1.46
P-10 Pd	9.5	31.8	2.57	3.7
P-15 Pd	9.3	123.7	0.66	14
P-20 Pd	8.8	240.2	0.34	26
P-30 Pd	8.2	388.9	0.21	38.8

## Data Availability

Data will be available on request.

## References

[1] Lopes A.C., Carabineiro S.A.C., Pereira M.F.R., Botelho G., Lanceros-Mendez S. (2013). Nanoparticle size and concentration dependence of the electroactive phase content and electrical and optical properties of Ag/poly(vinylidene fluoride) composites. Chem. Phys. Chem..

[2] Lv J. (2013). Effect of wettability on surface morphologies and optical properties of Ag thin films grown on glass and polymer substrates by thermal evaporation. Appli. Surf. Sci..

[3] Kalu E.E., Daniel M., Bockstaller M.R. (2012). Synthesis, characterization, Electrocatalyic and catalytic activity of thermally generated polymer-stabilized metal nanoparticles. Int. J. Electrochem. Sci..

[4] Aberg C.M., Seyam M.A., Lassell S.A., Bronstein L.M., Spontak R.J. (2008). In situ growth of Pd nanoparticles in crosslinked polymer matrices. Macromol. Rapid Commun..

[5] Darmadi I., Stolas A., Ostergren I., Berke B., Nugroho F.A.A., Minelli M., Lerch S., Tanyeli I., Lund A., Andersson O. (2020). Bulk-processed Pd nanocube-poly(methyl methacrylate) nanocomposites as plasmonic plastics for hydrogen sensing. Appl. Nano Mater..

[6] Troger L., Hunnefeld H., Nunes S., Oehring M., Fritsch D. (1997). Structural characterization of catalytically active metal nanoclusters in poly(amide imide) films with high metal loading. J. Phys. Chem..

[7] Yang X.J., He W., Wang S.X., Zhou G.Y., Tang Y. (2012). Preparation and properties of a novel electrically conductive adhesive using a composite of silver nanorods, silver nanoparticles, and modified epoxy resin. J. Mater. Sci. Mater. Electron..

[8] Hong M.C., Choi M.C., Chang Y.W., Lee Y., Kim J., Rhee H. (2012). Palladium nanoparticles on thermoresponsive hydrogels and their application as recyclable Suzuki-Miyaura coupling reaction catalysts in water. Adv. Synt. Catal..

[9] Pucci A., Boccia M., Galembeck F., Leite C.A.D.P., Tirelli N., Ruggieri G. (2008). Luminescent nanocomposites containing CdS nanoparticles dispersed into vinyl alcohol based polymers. React. Funct. Polym..

[10] Fateixa S., Girao A.V., Nogueira H.I.S., Trindade T. (2011). Polymer based silver nanocomposites as versatile solid film and aqueous emulsion SERS substrates. J. Mater. Chem..

[11] Dhakal T.R., Mishra S.R., Glenn Z., Rai B.K. (2012). Synergistic effect of PVP and PEG on the behavior of silver nanoparticle-polymer composites. J. Nanosci. Nanotech..

[12] Chatterjee U., Jewrajka S.K. (2007). Synthesis of block copolymer-stabilized Au6Ag alloy nanoparticles and fabrication of poly(methyl methacrylate)/Au-Ag nanocomposite film. J. Coll. Inter. Sci..

[13] Berta M., Loppinet B., Vlassopoulos D., Askounis A., Koutsos V., Pastoriza-Santos I., Liz-Marzan L.M. (2012). Tailoring the properties of grafted silver nanoprism composites. Polymer.

[14] Wu Y.S., Liao L.D., Pan H.C., He L., Lin C.T., Tan M.C. (2017). Fabrication and interfacial characteristics of surface modified Ag nanoparticle based conductive composites. RSC Adv..

[15] Molaba M.P., Dudic D., Luyt A.S. (2015). Influence of the presence of medium-soft paraffin wax on the morphology and properties of iPP/silver nanocomposites. Express Polym. Lett..

[16] Radad K., Al-Shraim M., Moldzio R., Rausch W.-D. (2012). Recent advances in benefits and hazards of engineered nanoparticles. Environ. Toxicol. Pharmacol..

[17] Thompson D., Kranbuehl D., Espuche E. (2016). Polymer Nanocomposite Film with Metal Rich Surface prepared by In Situ Single-Step Formation of Palladium Nanoparticles: An Interesting Way to Combine Specific Functional Properties. Nanomaterials.

[18] Simon S., Alcouffe P., Espuche E. (2014). Hybrid films of polyetherimide containing in situ grown Ag, Pd and AgPd alloy nanoparticles: Synthesis route, morphology, gas transport properties. J. Polym. Sci. Polym. Phys..

[19] Clémenson S., Espuche E., David L., Léonard D. (2010). Nanocomposite membranes of polyetherimide nanostructured with palladium particles: Processing route, morphology and functional properties. J. Membr. Sci..

[20] Clémenson S., Léonard D., Sage D., David L., Espuche E. (2008). Metal nanocomposite films prepared in situ from PVA and silver nitrate. Study of the nanostructuration process and morphology as a function of the in situ routes. J. Polym. Sci. Part. A Polym. Chem..

[21] Clémenson S., David L., Espuche E. (2007). Structure and morphology of nanocomposite films prepared from polyvinyl alcohol and silver nitrate: Influence of thermal treatment. J. Polym. Sci. Polym. Chem..

[22] Compton J.M., Thompson D.W., Kranbuehl D.E., Ohl S., Gain O., David L., Espuche E. (2006). Hybrid films of polyimide containing in situ generated silver or palladium nanoparticles: Effect of the particle precursor and of the processing conditions on the morphology and the gas permeability. Polymer.

[23] Cheviron P., Gouanvé F., Espuche E. (2014). Green synthesis of colloid silver nanoparticles and resulting biodegradable starch/silver nanocomposites. Carbohydr. Polym..

[24] Madhuri U.D., Saha J., Radhakrishnana T.P. (2018). ‘Dipcatalysts’ based on polymer-metal nanocomposite thin films: Combining soft-chemical fabrication with efficient application and monitoring. Chem. Nano Mater..

[25] Hariprasad E., Radhakrishnan T.P. (2013). In situ fabricated polymer-silver nanocomposite thin film as an inexpensive and efficient substrate for surface-enhanced raman scattering. Langmuir.

[26] Rybak A., Boiteux G., Melis F., Seytre G. (2010). Conductive polymer composites based on metallic nanofiller as smart materials for current devices. Comp. Sci. Techn..

[27] Sheth J.P., Xu J., Wilkes G.L. (2003). Solid state structure-property behavior of semicrystalline poly(ether-block-made) PEBAX® thermoplastic elastomers. Polymer.

[28] Boulares A., Tessier M., Maréchal E. (2000). Synthesis and characterization of poly(copolyethers-block-polyamides) II. Characterization and properties of the multiblock copolymers. Polymer.

[29] Barbi V., Funari S.S., Gehrke R., Scharnagl N., Stribeck N. (2003). SAXS and the gas transport in polyether-block-polyamide copolymer membranes. Macromolecules.

[30] Magana S., Gain O., Gouanvé F., Espuche E. (2016). Influence of different alkyl-methylimidazolium tetrafluoroborate ionic liquids on the structure of PEBAX films. Consequences on thermal, mechanical and water sorption and diffusion properties. J. Polym. Sci. Polym. Phys..

[31] Sabard M., Gouanvé F., Espuche E., Fulchiron R., Seytre G., Trouillet-fonti L., Fillot L.-A. (2014). Influence of montmorillonite and film processing conditions on the morphology of polyamide 6: Effect on ethanol and toluene barrier properties. J. Membr. Sci..

[32] Samet M., Levchenko V., Boiteux G., Seytre G., Kallel A., Serghei A. (2015). Electrode polarization vs. Maxwell-Wagner-Sillars interfacial polarization in dielectric spectra of materials:characteristicfrequencies and scaling laws. J. Chem. Phys..

[33] Murugendrappa M.M., Khasim S., Ambika Prasad M.V.N. (2000). Conductivity and DSC studies of poly(ethylene glycol) and its salt complexes. Indian J. Eng. Mater. Sci..

[34] Zhao B., Zhang X., Deng J., Zhang C., Li Y., Guo X., Zhang R. (2020). Flexible Pebax/graphene electromagnetic shielding composite films with a negative pressure effect of resistance for pressure sensors applications. RSC Adv..

